# Family Spillovers and Long-Term Care Insurance

**DOI:** 10.1093/geroni/igaa057.2396

**Published:** 2020-12-16

**Authors:** Norma Coe, Courtney Van Houtven, Gopi Goda

**Affiliations:** 1 University of Pennsylvania, Philadelphia, Pennsylvania, United States; 2 US Department of Veterans Affairs, Durham, North Carolina, United States; 3 Stanford University, Stanford, Colorado, United States

## Abstract

We examine how the presence of long-term care insurance (LTCI) spills over to family outcomes, including informal care, co-residence, and labor supply of adult children. We instrument for long-term care insurance with changes in state tax policies to address the endogeneity of LTCI coverage. We find that for tax-filing families in the top third of the income distribution, LTCI coverage leads to a 50 percent reduction the parents’ perceptions of the willingness of people to care for them in the future, including their adult children. We also find that LTCI causes changes in the residential decisions of adult children, with lower co-residence rates and higher likelihood of living within 10 miles of the parent. We also find small decreases in part-time work among adult children. Our findings provide empirical support for the presence of family spillovers of LTCI on the economic behaviors of family members. Part of a symposium sponsored by the Economics of Aging Interest Group.

